# Fish polyomaviruses belong to two distinct evolutionary lineages

**DOI:** 10.1099/jgv.0.001041

**Published:** 2018-03-08

**Authors:** Koenraad Van Doorslaer, Simona Kraberger, Charlotte Austin, Kata Farkas, Melissa Bergeman, Emma Paunil, William Davison, Arvind Varsani

**Affiliations:** ^1^​ School of Animal and Comparative Biomedical Sciences, University of Arizona, 1657 E Helen St., Tucson, AZ 85721, USA; ^2^​ Cancer Biology Graduate Interdisciplinary Program, Genetics Graduate Interdisciplinary Program, Bio5 Institute, and the University of Arizona Cancer Center University of Arizona, 1657 E Helen St., Tucson, AZ 85721, USA; ^3^​ The Biodesign Center for Fundamental and Applied Microbiomics, Center for Evolution and Medicine and School of Life Sciences, Arizona State University, Tempe, AZ 85287, USA; ^4^​ School of Biological Sciences, University of Canterbury, Christchurch, New Zealand; ^5^​ School of Environment, Natural Resources and Geography Bangor University Bangor, LL57 2UW, UK; ^6^​ Structural Biology Research Unit, Department of Clinical Laboratory Sciences, University of Cape Town, Cape Town, 7925, South Africa

**Keywords:** *Polyomaviridae*, Antarctica, emerald notothen, *Trematomus bernacchii*

## Abstract

The *Polyomaviridae* is a diverse family of circular double-stranded DNA viruses. Polyomaviruses have been isolated from a wide array of animal hosts. An understanding of the evolutionary and ecological dynamics of these viruses is essential to understanding the pathogenicity of polyomaviruses. Using a high throughput sequencing approach, we identified a novel polyomavirus in an emerald notothen (*Trematomus bernacchii*) sampled in the Ross sea (Antarctica), expanding the known number of fish-associated polyomaviruses. Our analysis suggests that polyomaviruses belong to three main evolutionary clades; the first clade is made up of all recognized terrestrial polyomaviruses. The fish-associated polyomaviruses are not monophyletic, and belong to two divergent evolutionary lineages. The fish viruses provide evidence that the evolution of the key viral large T protein involves gain and loss of distinct domains.

## Introduction

The *Polyomaviridae* is a family of circular dsDNA viruses with an average genome size of about 5000 base pairs [1]. The polyomavirus (PyV) genomes are packed into ~45 nm icosahedral particles made up of three structural proteins (VP1, VP2 and VP3) [2]. The PyV genomes are transcribed bidirectionally, with the structural genes being coded on one strand and the large and small tumour antigen (T-Ag and t-Ag) on the complementary strand.

PyV infections have been described in humans and a wide array of animals. The first PyV was isolated from a murine tumour [3]. PyVs infect the urinary, digestive and respiratory tracts, as well as the skin [4]. Since the discovery of the Merkel cell PyV, the causative agent of the rare, aggressive Merkel cell carcinoma, many novel (human) PyVs have been identified and described [5, 6]. Specifically, technical improvements increased both the number of genomes being identified, as well as the diversity of PyV-associated animal hosts [7–9]. PyVs are classified into four genera (*Alpha*-, *Beta*-, *Gamma*- and *Deltapolyomavirus*). Members of the *Gammapolyomavirus* are found exclusively in birds [2]. Recently, diverse PyVs were identified in various fish species [2, 10], and these remain unclassified. Similar to papillomaviruses [11], PyVs appear to be highly species-specific [10]. Traditionally, PyVs were believed to have coevolved with amniotes. However, to understand PyV evolution and disease, a more comprehensive view of viral ecology and diversity is needed.

As part of an ongoing study on viral diversity associated with animals of the Antarctic, we identified a novel PyV in an emerald notothen (*Trematomus bernacchii*). We analysed the complex evolutionary history of this novel and other fish-associated PyVs.

## Results and discussion

### Fish-associated PyVs

Using a metagenomics approach, we recovered a novel circular dsDNA genome of 5541 bp (GenBank accession # MG800627) from a liver sample of an emerald notothen (*T. bernacchii*). *T. bernacchii* is a typical notothenioid in that it is benthic, territorial and does not move great distances. The species is restricted to coastal Antarctica where temperature changes little from −1.9 °C throughout the year. It is worth commenting that the species is a relatively modern perciform fish. The notothenioids have been isolated for at least 35 million years, associated with cooling of Antarctic waters, while the genus Trematomus is relatively new, originating in Antarctica 5–10 million years ago [12].

Very few viruses have been described from Antarctic (marine) animals [13]. Initial analysis of this putative new virus resulted in the identification of ORFs with similarity to the PyV T-Ag and structural proteins (VP1 and VP2), suggesting that we recovered the genome of a new PyV associated with fish. We propose to name this novel PyV *T. bernacchii* PyV type 1 (TbPyV1). PyVs were long believed to only infect amniotes. However, recent technological advances, such as high-throughput sequencing, have identified a number of PyVs associated with fish. The first PyV from the Perciformes, or ‘perch-like’ group of bony fish, was isolated from a black sea bass *(Centropristis striata).* In addition to the BassPyV [14], PyVs have been isolated from a gilt-head sea bream (*Sparus aurata*) and a sharp-spined notothen (*Trematomus pennellii*). A PyV was characterized from a cartilaginous Guitarfish (*Rhynchobatus djiddensis*) [10, 15]. Furthermore, hybrid viral genomes containing an apparent homologue of the T-Ag protein have been described in two separate species of eels, *Anguilla japonica* [16], and *Anguilla marmorata* [17, 18].

### Fish PyVs are highly divergent

**Fig. 1.**
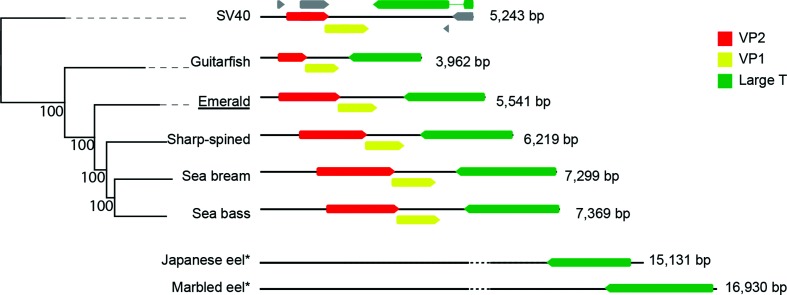
Genome structure of the fish-associated PyVs. Neighbour-joining (NJ) phylogenetic tree inferred from alignments of the complete viral genome sequences of the known fish PyVs and SV40. English host names are used to refer to the associated fish PyVs. The novel virus identified in this work is underlined. Numbers at the nodes represent the bootstrap support for the NJ tree. To the right of the tree the (linearized) genome organization for each genotype is shown, highlighting the VP2 (red), VP1 (yellow) and T-Ag (green) ORFs. Numbers refer to the genome size. Grey ORFs in the SV40 diagram refer to (from left to right) Agno, VP3, Alto and t-Ag. The eel-associated viruses are also shown for reference (Fig. S1, available in the online version of this article shows fully annotated versions of the eel virus genomes).

**Fig. 2.**
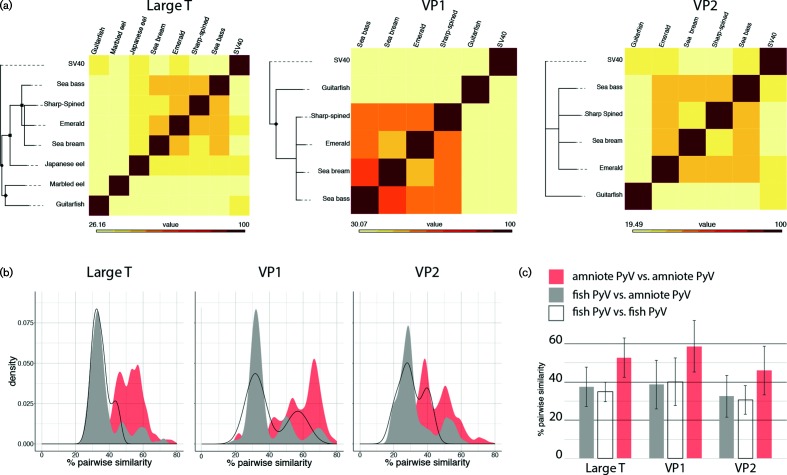
Fish viral proteins are evolutionarily divergent (a) maximum likelihood phylogenetic trees of individual protein trees. English host names are used to refer to the fish-associated PyVs. Trees were rooted using SV40, and the remaining (*n*=114) amniote PyVs were removed to improve visualization (complete trees available in Fig. S2). Symbols indicate bootstrap support (square=100; diamond >90; circle >80). Nodes with bootstrap support less than 70 % were collapsed. Heat maps to the right of the tree show the pairwise sequence similarity. (b) Graphs comparing the percentage pairwise similarity of different PyV proteins. The black line shows the distribution of pairwise comparisons of fish PyVs. The grey plot represents comparisons between fish and amniote PyV, while the red plots show comparisons between amniote PyV. This data shows that amniote PyVs are more similar to other amniote PyVs than fish PyVs are to each other. (c) Bar graphs showing average and sd of the curves in (b).

The genome sizes of the five fish PyVs are highly variable, with genome sizes ranging from 3962 to 7369 bp (Fig. 1). Fish PyVs have both the smallest and largest genomes of all known PyVs which typically are about 5 kb in length [19, 20]. All canonical fish PyVs contain ORFs that are predicted to be homologous to the T-Ag, VP1 and VP2 proteins. Remarkably, these proteins are highly divergent (Fig. 2), and they share no more than 46, 62 and 42 % amino-acid sequence similarity, respectively (Fig. 2a). When comparing pairwise sequence similarity, the individual fish PyVs are as divergent from each other, as they are to any of the other (amniotic) PyVs (Fig. 2b). Members of a PyV genus typically share around 60 % T-Ag amino-acid sequence similarity. In fact, the lowest pairwise identity between individual PyV species belonging to the same viral genus is 47.8 % (Hamster and Straw Colored Fruit bat PyVs). The two most closely related fish PyVs (sea bass and emerald notothen PyV) share only 46 % similarity. According to the current PyV classification [2], each known fish-associated PyV occupies a PyV distinct genus. The current data suggests that this suggests that fish PyVs diverge at a faster rate than amniote PyVs, but additional fish-associated PyVs need to be characterized to properly resolve this question.

### Cartilaginous and perciform fish PyVs belong to distinct evolutionary lineages

**Fig. 3.**
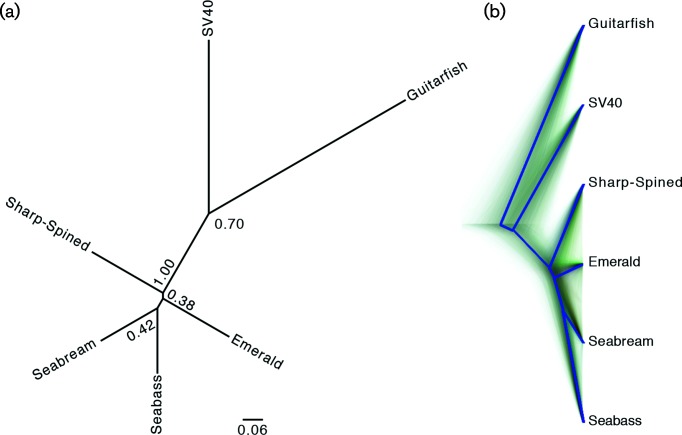
Species tree of the fish-associated PyVs. (a) Unrooted Bayesian species tree was based on the T-Ag, VP1 and VP2 protein sequences. Nodes with posterior support less than 0.7 were collapsed. English host names are used to refer to the associated fish PyVs. (b) DensiTree representation of the tree in (a) shows the highly uncertain evolutionary history of the perciform fish PyVs. The solid blue line represents the root canal, which helps to focus on the main features of the tree set.

To investigate the evolutionary relationships between the distinct Eugnathostomata (fish, amphibians, reptiles and mammals) PyVs we constructed four distinct phylogenetic trees. The individual protein sequences [T-Ag (*n*=122; 115 amniote, five fish and two eel viruses), VP1 (*n*=120) and VP2 (*n*=120)] were aligned, and used to estimate maximum likelihood phylogenetic trees. To facilitate visualization, these trees were rooted on the clade containing SV40, one of the most studied PyVs, after which all non-SV40 leaves were culled (Fig. 2a). The full, non-culled trees are available in Fig. S2. In addition to the individual protein trees, we also constructed a species tree (Fig. 3). The maximum likelihood trees based on individual proteins (Figs 2 and S2) support that the fish-associated PyVs cluster separately from the amniote PyVs. For computational reasons associated with the species tree construction, we used the well-studied SV40 sequence to represent the non-fish PyV cluster. Overall, the four trees (three protein trees and one species tree) show that there is a lack of resolution within the perciform fish PyV clade. This analysis suggests that the perciform and cartilaginous fish PyVs are not monophyletic. Furthermore, the current PyV phylogenetic tree can be divided into three major clades (Fig. 3). One clade contains the current perciform-fish-associated PyVs; the second clade contains the cartilaginous guitarfish PyV as a sole representative. The third main clade consists of all the currently recognized (amniote) PyV genera [2, 10] (Fig. 3). It remains to be determined whether the genomic diversity within the fish clades will be similar to what is seen in the amniotic clade. A recent report describes a PyV isolated from a rectal swab of a Pomona Leaf-Nosed Bat (*Hipposideros pomona*) [21]. This genome was published after we began our analysis, and it was therefore not included in our analysis, this bat-associated PyV clusters within the bony-fish-associated PyV clade [21]. It is to be determined whether the isolation of this virus represents an active infection or a dietary/environmental origin. Nonetheless, the PyV taxonomy appears to have three major, divergent clades (Fig. 3). The related papillomavirus taxonomy recently proposed the use of sub-families to refer to these major, highly divergent clades (https://talk.ictvonline.org/files/proposals/animal_dna_viruses_and_retroviruses/m/animal_dna_under_consideration/6943).

### Fish PyVs have unique T-Ag proteins

**Fig. 4.**
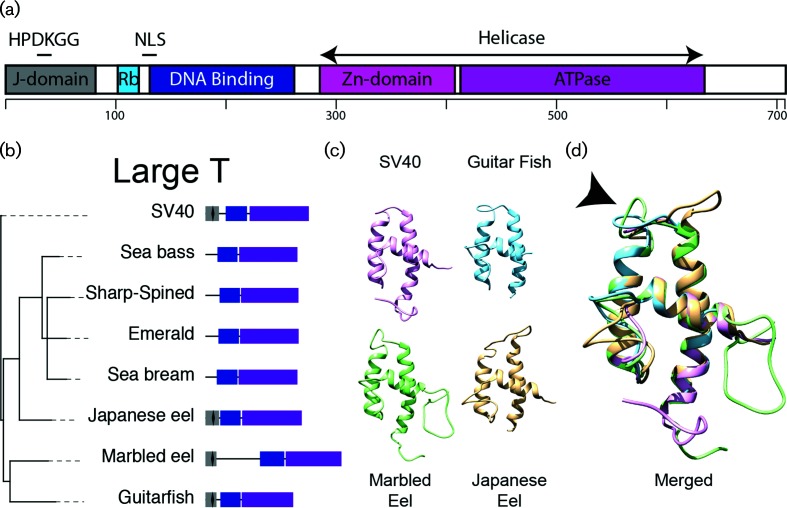
Evolutionary analysis of the T-Ag protein. (a) Diagram of the SV40 T-Ag protein (708 aa). The J-domain, with embedded HPDKGG hexapeptide, Rb binding motif, nuclear localization signal (NLS), and helicase domains are indicated. The helicase domain is divided into a zinc (Zn) domain and the ATPase. (b) Maximum likelihood phylogenetic tree of the T-Ag protein. Trees were rooted using SV40, and the remaining (*n*=114) amniote PyVs were removed to improve visualization (complete trees available in Fig. S2). Nodes with bootstrap support less than 70 % were collapsed. Diagrams to the right of the tree show the individual domains in T-Ag DnaJ (grey), the HPDKGG hexapeptide (black diamond). Origin binding domain (blue) and helicase domain (purple). English host names are used to refer to the associated fish PyVs. (c) Homology models of the different PyV DnaJ domains homology modelled on the murine PyV DnaJ domain (1FAF [42]). Models for SV40 (purple), the guitarfish (blue), marbled (green) and Japanese (yellow) eel viruses were visualized and analysed using Chimera version 1.11.2 [43]. (d) Merged overlay of the models shown in (c). The black arrow head points towards the conserved HPDKGG hexapeptide.

In SV40 and other canonical PyVs, the T-Ag protein can be divided into three domains. The N-terminal end of canonical T-Ag proteins contains the DnaJ domain [22], followed by the origin binding domain, and finally the core PyV helicase/ATPase domain (Fig. 4). The DnaJ domain is made up of a short CR1 (13–17 aa) motif and a conserved HPDKGG hexapeptide sequence. The approximately 70 amino-acid residue-long DnaJ domain is a defining feature of the Hsp40 family of molecular chaperones that coordinate substrate selection of the Hsp70 chaperone machine [23]. Specifically, the HPDKGG motif is important for Hsc70 binding [24], and efficient SV40 viral replication *in vitro* [25]. The DnaJ motif is followed by the conserved Rb-binding (LxCxE) motif. The interaction between T-Ag and pRB disrupts E2F-mediated transcription, thus driving cells into S-phase. The T-Ag origin binding domain is responsible for recognition and binding to the PyV origin of replication. Finally, the core PyV helicase/ATPase domain, which contains the hallmark Walker (ATPase) motif, is important for T-Ag replication [26] (Fig. 4a). Within the fish PyVs, the origin binding domain and helicase motifs are fairly conserved, but the perciform fish PyVs T-Ag proteins do not contain a DnaJ domain. This DNaJ domain is present in the cartilaginous Guitarfish PyV, and in the eel-associated viruses (Fig. 4b). Indeed, homology-based modelling confirms that the DnaJ domain of the guitarfish and both eel viruses adopts a fold similar to the fold observed in SV40 (Fig. 4c). The arrow in Fig. 4(d) indicates the location of the conserved HPDKGG hexapeptide sequence. Computational approaches did not recover any (structural) similarity between the bony fish T-Ag and known DnaJ domains. Together with the phylogenetic tree shown in Fig. 3(a), this suggests that the T-Ag protein lost the DnaJ motif after perciform fish diverged. On the other hand, the canonical pRb interaction motif (LxCxE) is not present in any of the fish T-Ag proteins and appears to be unique to the extant amniote PyVs. Suggesting that the LxCxE (and associated ability to interact with pRb) may have evolved uniquely during the evolution of the extant amniote PyVs, following their divergence from fish PyVs. Finally, prototypical PyVs use alternative splicing of early pre-mRNA to generate the T-Ag and t-Ag mRNAs (Fig. 1). In T-Ag, this splicing event connects the N-terminal DnaJ domain just upstream of the conserved LxCxE motif, while the small t-Ag protein consists of the DnaJ motif followed by a unique sequence. The associated splice donor and acceptor sites do not appear to be conserved in the fish-associated early mRNAs. Consequently, a t-Ag ORF does not appear to be present in these viruses.

The evolutionary history of the T-Ag protein is complicated by the existence of a scorpion PyV [10]. The putative T-Ag protein of this virus contains both a DnaJ domain and a LxCxE pRb interaction motif. According to the hypothesis of cospeciation, the Arachnid and *Eugnathostomata* PyVs should have diverged at the same time as the hosts. The scorpion viruses were assembled based on reads mined from the whole genome shotgun database (contig AXZI01204118) supplemented with individual reads from the parent Sequence Read Archive (SRA) dataset [10]. PyVs have been proposed to recombine more readily than the related papillomaviruses (see next paragraph and [10]). We propose that the scorpion PyV may represent an ancient recombination event where the structural VP1 and VP2 proteins from an ancestral arthropod virus hybridized with the T-Ag cassette of an ancestral virus.

### Inter-family recombination

Phylogenetic trees based on individual viral proteins are not congruent with each other [10]. It has been proposed that intra-host divergence and recombination play important roles in the evolution of extant PyVs [10]. In the absence of the parent genomes, it is often hard to provide evidence of recombination between closely related viruses. However, PyVs appear to have recombined with viruses belonging to distinct viral families. Both rel-associated viruses [17, 18] contain a protein that is homologous to the PyV T-Ag protein (Figs 1, 2 and 4). In addition to T-Ag, these viruses contain four ORFs (~4 000 bp total; Fig. S1) that are highly conserved in both viruses but have no other known homologues. The remaining ~10 000 bp is unique to each virus and does not have homologues in the GenBank database. Remarkably, the Japanese and marbled eel virus T-Ag protein are not monophyletic (Figs 2a and 4), suggesting that the eel viruses are the result of two separate recombination events between a PyV and an as yet unknown family of viruses. Based on the presence of a DnaJ domain, these separate recombination events likely preceded the divergence of perciform fish from eels, roughly 275 million years ago. A third inter-family recombination event involves a papillomavirus. A western barred bandicoot (*Perameles bougainville*) virus contains a PyV T-Ag and small T cassette combined with the L1 and L2 papillomavirus structural genes. In a T-Ag phylogenetic tree (Supplementary data), the bandicoot T-Ag is sister to a clade containing PyVs infecting birds, suggesting a common ancestor between these proteins. The bandicoot viruses L1 and L2 proteins are related to a known marsupial-tropic papillomavirus [27, 28].

### Concluding remarks

In recent years, viral discovery, facilitated by metagenomics and high-throughput sequencing, has enabled the identification of novel viruses in the family *Polyomaviridae*. Based on the available sequence data we suggest that PyVs belong to three distinct evolutionary lineages, two of which were made up of fish-associated viruses. The present study offers a detailed look at these highly diverse fish PyVs and provides insight into the evolution of the viral large T protein. Specifically, the C-terminal portion of this critical viral protein shows evidence for evolutionary specialization. The LT antigen of a subset of fish PyVs lost the prototypical DnaJ domain and replaced it with a domain of unknown function. On the other hand, the canonical pRb interaction motif, which is located just downstream of the DnaJ motif, may be unique to amniote PyVs. The LT antigen is central to the viral life cycle, and understanding its evolutionary history, should provide further insights into the function of the protein.

## Methods

### Sampling and sample processing

An emerald notothen individual was sub-sampled as part of a *Trematomus* spp study in the Ross Sea (Antarctica) during the 2012–2013 summer field season. Approximately 1 cm^3^ of the emerald notothen stomach and liver were separately homogenized in 20 ml of SM buffer (0.1M NaCl, 50 mM Tris/HCl – pH 7.4, 10 mM MgSO_4_) using a mortar and pestle. The homogenates were centrifuged at 6000 ***g*** for 10 min to pellet cellular debris, and the supernatant was then filtered through 0.22 µm syringe filters. The filtrates were made up to 10 % w/v with PEG 8000 to precipitate viral particles (6000 ***g*** for 20 min). The resulting precipitates were resuspended in 1 ml of SM buffer. In total, 200 µl of this was subsequently used to extract viral DNA using the High Pure Viral Nucleic Acid Kit (Roche Diagnostics, USA). Viral DNA (1 µl) from each of the two tissue types was used as a template to amplify circular DNA molecules using rolling-circle amplification (RCA) with the TempliPhi kit (GE Healthcare, USA).

### Sequencing of novel PyV from *T. bernacchii*

The RCA DNA (20 µl aliquot from each sample) was sequenced on an Illumina 4000 (Illumina) sequencer at Macrogen (Korea). The paired-end reads were *de novo* assembled using ABySS v2.02 [29], and contigs >250 nucleotides were analysed using blastx [30] against a local viral database. A contig of 372 nucleotides was identified in the liver sample with similarities to VP2 and VP1 of fish PyVs. Based on the sequence of this contig, a pair of abutting primers (F: 5′-CGCTGCTAAAGGAAATAAAATCAAGATATGGGAAT-3′; R: 5′-ACTGGAATTTTAGGCAAATCTATAGCTGACGTTAG-3′) were designed to recover the full genome of this putative PyV. A 0.5 µl sample of the RCA reaction was used as a template for PCR amplification using the abutting primers and Kapa HiFi Hotstart DNA polymerase with the following thermal cycling protocol: 95 °C for 3 min; 25 cycles of 98 °C for 20 s, 60 °C for 15 s, 72 °C for 5 min, and a final extension of 72 °C for 6 min. The amplicon was resolved on a 0.7 % agarose gel, the ~5 kb product was excised, gel-purified and cloned into the pJET1.2 plasmid vector (ThermoFisher, USA). The recombinant plasmid was Sanger-sequenced by primer walking at Macrogen (Korea), and the Sanger sequence contigs were assembled using DNA Baser V4 (Heracle BioSoft S.R.L., Romania).

### Phylogenetic analysis

Annotated PyV genomes were downloaded from https://home.ccr.cancer.gov/Lco/PyVE.asp. The master file was filtered for viruses that contained (annotated) VP1, VP2 and T-Ag ORFs. The final dataset contained 122 T-Ag, 120 VP1 or 120 VP2 gene sequences. ORFs were translated into putative proteins and aligned using the l-INS-i option of mafft [31]. ProtTest (version 3.4.2; [32, 33]) identified the LG+I+G+F model of evolution [34] for each of the three proteins. Maximum likelihood (ML) trees were inferred using RAxML-HPC v.8 on XSEDE [35], performing a rapid bootstrap analysis followed by a search for the best tree. The number of bootstrap replicates was determined according to the autoMRE criteria implemented in RAxML [36, 37]. The optimal ML tree was rooted on the clade holding SV40 [10], and the resulting tree was trimmed to remove the non-fish PyVs.

The T-Ag, VP1 and VP2 genes from the distinct fish viruses and SV40 were aligned as described above. These gene alignments were used to construct a species tree for these viruses. *beast as implemented in beast v.2.47 on XSEDE [35] was used. The same LG model as above was used to estimate the individual gene trees. A relaxed molecular clock implementing the ‘linear with constant root’ multispecies coalescent function was used to estimate the rate of sequence evolution. The MCMC chains were run for 1×10^8^ generations. Following a 1 % pre-burnin, samples were collected every 5000th generation. A 10 % burnin was discarded, an estimated sample size of >200 was achieved for most parameters. Post-burnin trees were loaded into DensiTree for visualization [38, 39].

### Homology modelling

pGenTHREADER and pDomTHREADER as implemented on the psipred server were used to identify structural homology between the N-terminal (DnaJ) domain of each of the fish PyVs [40, 41]. Using the guitarfish, and both eel viruses the psipred algorithm identified the murine PyV DnaJ domain (1FAF [42]) as one of the top three hits. 1FAF was used to model the DnaJ motif of these fish-associated large T proteins. Importantly, the other fish-associated PyVs did not return any significant hits with the DnaJ motif. Homology models were visualized and analysed using Chimera version 1.11.2 [43].

## Supplementary Data

1667 Supplementary File 2
