# Supplementary material for: Fish polyomaviruses belong to two distinct evolutionary lineages
Source: J Gen Virol. 2018 Mar 8;99(4):567–73. doi: 10.1099/jgv.0.001041 (PMC5982132; doi:10.1099/jgv.0.001041)
Supplement: Supplementary File 2 [file jgv-99-567-s001.pdf]

**Supplemental material associated with Van Doorslaer et al., 2018**

**Supplemental Figure 1 Comparison of the eel associated viruses**

(A) Genome cartoon of the Marbled and Japanese eel viruses. The T-Ag like ORF is highlighted in green. The conserved L05, L06, L07, and L08 ORFs are shown in yellow. ORFs (larger than 500 nt) unique to each virus are identified in orange.

(B) graphical representation of the regions of similarity between the L05, L06, L07, and L08 proteins of the Japanese and Marbled eel viruses. Blots were generated using dotmatcher (EMBOSS 6.5.7 and [1,2]) as implemented in Geneious 10.2.2 [3]. The BLOSUM62 matrix with window size = 20, and Threshold = 23 was used.

**Supplemental Figure 2 Comparison of the eel associated viruses**

Maximum likelihood phylogenetic trees of individual protein trees (A) T-Ag, (B) VP1, and (C) VP2. The trees were rooted using the amniote PyV clade. These trees represent the unrooted, non-pruned versions of the trees in figure 2.

1. **Hancock JM, Bishop MJ**. EMBOSS (The European Molecular Biology Open Software Suite). In: *Dictionary of Bioinformatics and Computational Biology*. 2004.

2. **Rice P, Longden I, Bleasby A**. EMBOSS: the European Molecular Biology Open Software Suite. *Trends Genet* 2000;16:276–277.

3. **Kearse M, Moir R, Wilson A, Stones-Havas S, Cheung M, *et al.*** Geneious Basic: An integrated and extendable desktop software platform for the organization and analysis of sequence data. *Bioinformatics* 2012;28:1647–1649.

Figure S1

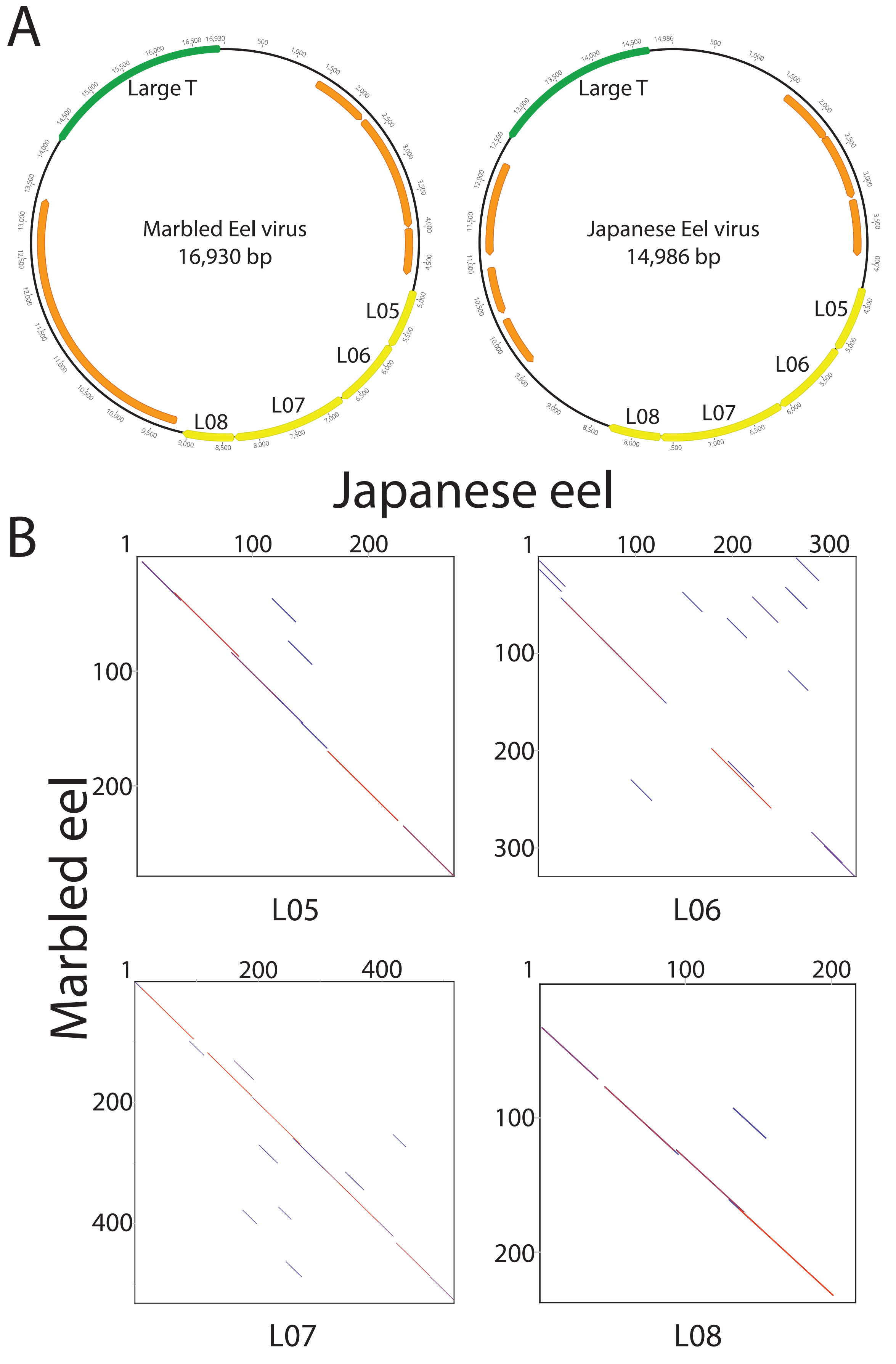
A
Large T
Marbled Eel virus
16,930 bp
L05
L06
L07
L08
Large T
Japanese Eel virus
14,986 bp
L05
L06
L07
L08
Japanese eel
B
Marbled eel
L05
L06
L07
L08

Figure S2A

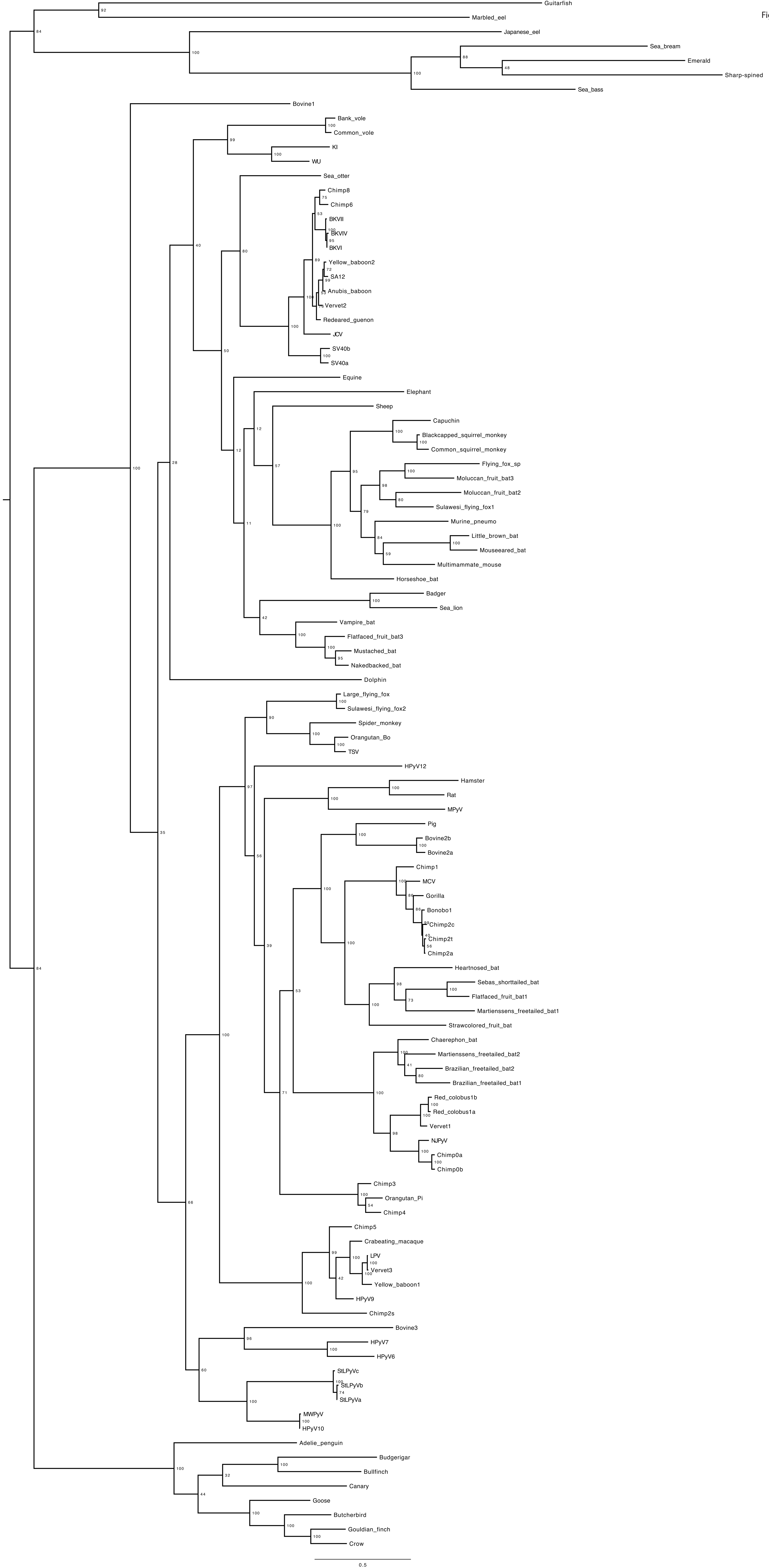
Guitarfish
Marbled_eel
Japanese_eel
Sea_bream
Emerald
Sharp-spined
Sea_bass
Bovine1
Bank_vole
Common_vole
KI
WU
Sea_otter
Chimp8
Chimp6
BKVII
BKVIV
BKVI
Yellow_baboon2
SA12
Anubis_baboon
Vervet2
Redeared_guenon
JCV
SV40b
SV40a
Equine
Elephant
Sheep
Capuchin
Blackcapped_squirrel_monkey
Common_squirrel_monkey
Flying_fox_sp
Moluccan_fruit_bat3
Moluccan_fruit_bat2
Sulawesi_flying_fox1
Murine_pneumo
Little_brown_bat
Mouseeared_bat
Multimammate_mouse
Horseshoe_bat
Badger
Sea_lion
Vampire_bat
Flatfaced_fruit_bat3
Mustached_bat
Nakedbacked_bat
Dolphin
Large_flying_fox
Sulawesi_flying_fox2
Spider_monkey
Orangutan_Bo
TSV
HPyV12
Hamster
Rat
MPyV
Pig
Bovine2b
Bovine2a
Chimp1
MCV
Gorilla
Bonobo1
Chimp2c
Chimp2t
Chimp2a
Heartnosed_bat
Sebas_shorttailed_bat
Flatfaced_fruit_bat1
Martienssens_freetailed_bat1
Strawcolored_fruit_bat
Chaerephon_bat
Martienssens_freetailed_bat2
Brazilian_freetailed_bat2
Brazilian_freetailed_bat1
Red_colobus1b
Red_colobus1a
Vervet1
NJPyV
Chimp0a
Chimp0b
Chimp3
Orangutan_Pi
Chimp4
Chimp5
Crabeating_macaque
LPV
Vervet3
Yellow_baboon1
HPyV9
Chimp2s
Bovine3
HPyV7
HPyV6
StLPyVc
StLPyVb
StLPyVa
MWPyV
HPyV10
Adelie_penguin
Budgerigar
Bullfinch
Canary
Goose
Butcherbird
Gouldian_finch
Crow
0.5

Figure S2B

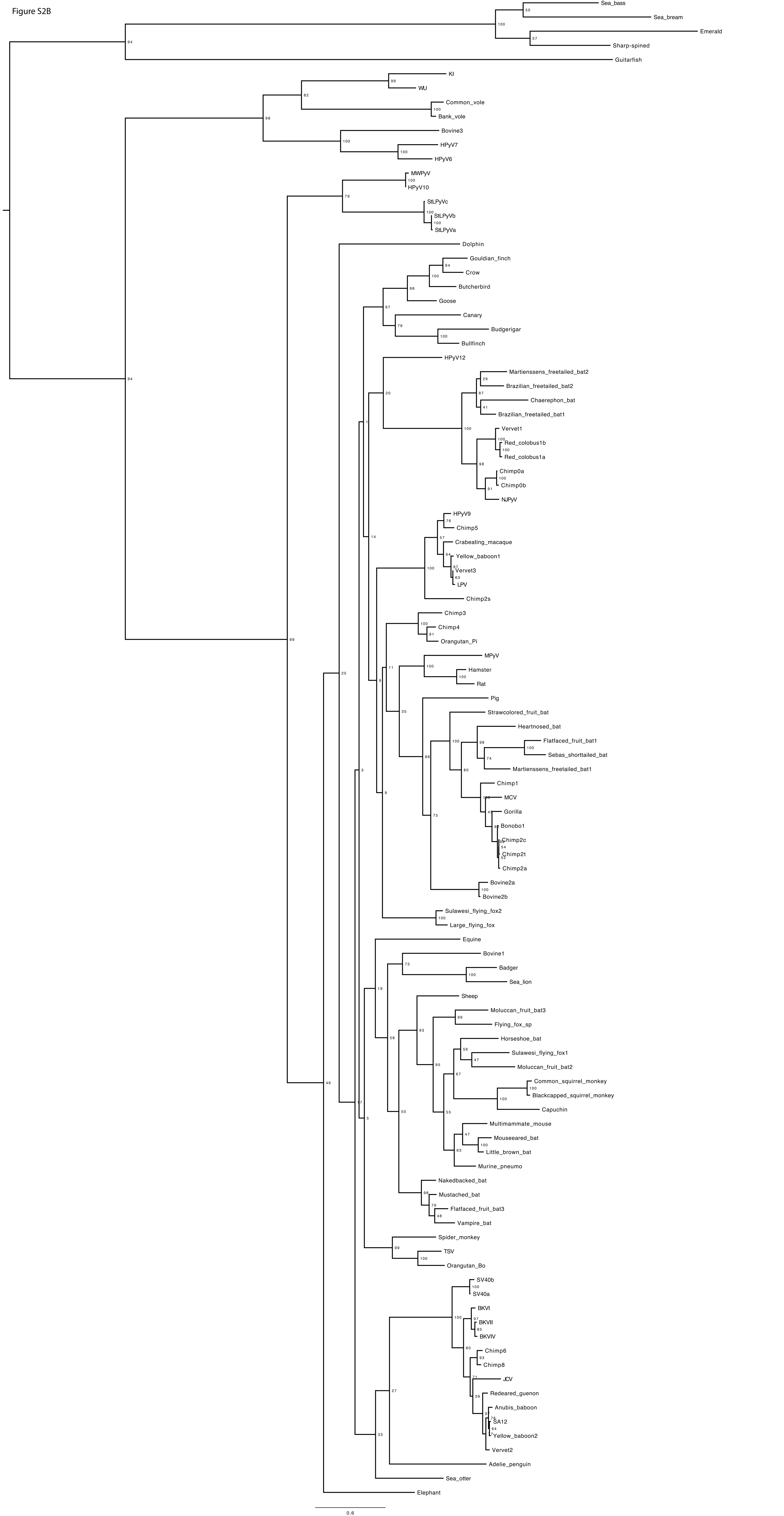
Sea_bass
Sea_bream
Emerald
Sharp-spined
Guitarfish
KI
WU
Common_vole
Bank_vole
Bovine3
HPyV7
HPyV6
MWPyV
HPyV10
StLPyVc
StLPyVb
StLPyVa
Dolphin
Gouldian_finch
Crow
Butcherbird
Goose
Canary
Budgerigar
Bullfinch
HPyV12
Martienssens_freetailed_bat2
Brazilian_freetailed_bat2
Chaerephon_bat
Brazilian_freetailed_bat1
Vervet1
Red_colobus1b
Red_colobus1a
Chimp0a
Chimp0b
NJPyV
HPyV9
Chimp5
Crabeating_macaque
Yellow_baboon1
Vervet3
LPV
Chimp2s
Chimp3
Chimp4
Orangutan_Pi
MPyV
Hamster
Rat
Pig
Strawcolored_fruit_bat
Heartnosed_bat
Flatfaced_fruit_bat1
Sebas_shorttailed_bat
Martienssens_freetailed_bat1
Chimp1
MCV
Gorilla
Bonobo1
Chimp2c
Chimp2t
Chimp2a
Bovine2a
Bovine2b
Sulawesi_flying_fox2
Large_flying_fox
Equine
Bovine1
Badger
Sea_lion
Sheep
Moluccan_fruit_bat3
Flying_fox_sp
Horseshoe_bat
Sulawesi_flying_fox1
Moluccan_fruit_bat2
Common_squirrel_monkey
Blackcapped_squirrel_monkey
Capuchin
Multimammate_mouse
Mouseeared_bat
Little_brown_bat
Murine_pneumo
Nakedbacked_bat
Mustached_bat
Flatfaced_fruit_bat3
Vampire_bat
Spider_monkey
TSV
Orangutan_Bo
SV40b
SV40a
BKVI
BKVII
BKVIV
Chimp6
Chimp8
JCV
Redeared_guenon
Anubis_baboon
SA12
Yellow_baboon2
Vervet2
Adelie_penguin
Sea_otter
Elephant
0.6

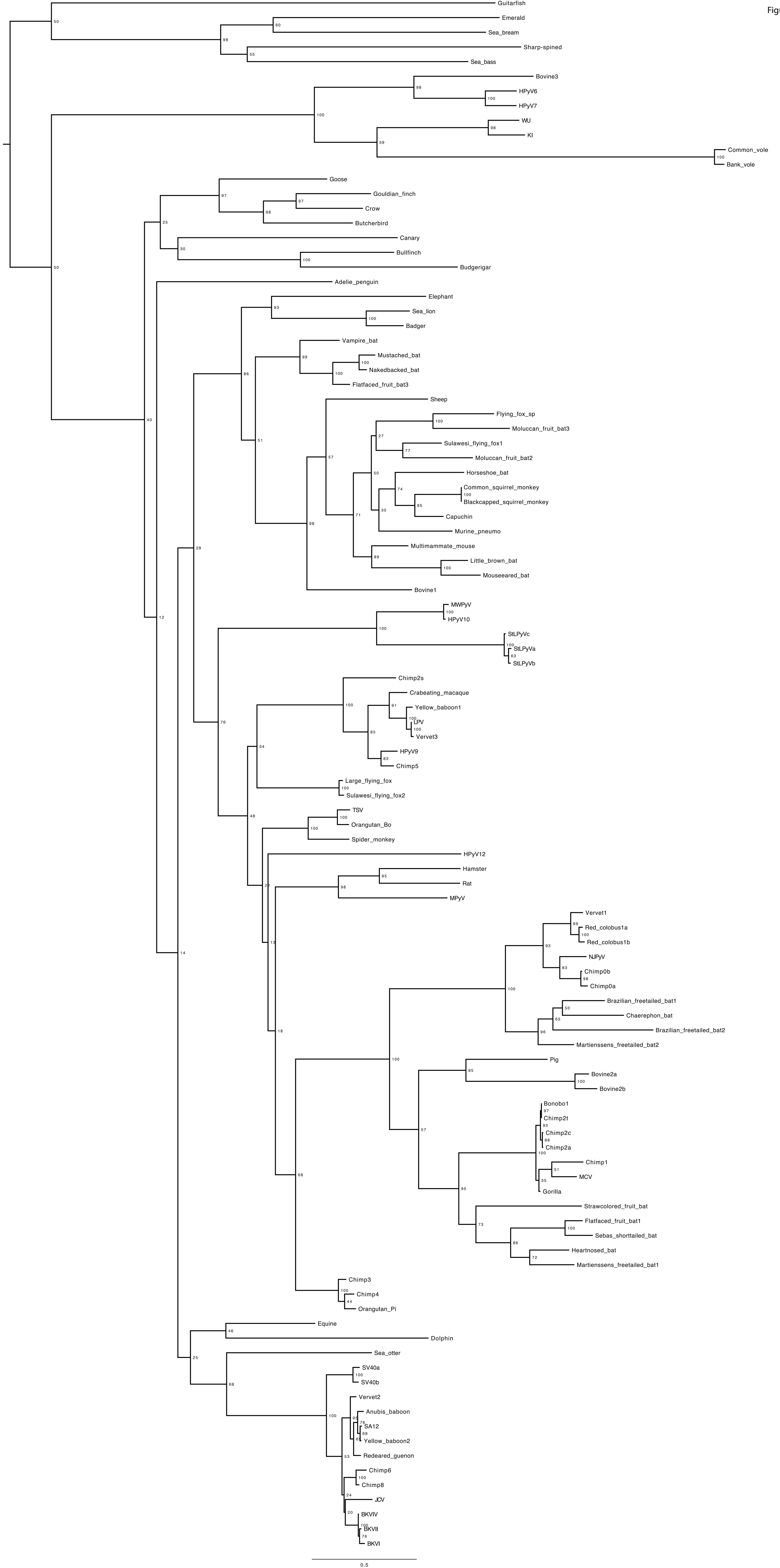
Guitarfish
Emerald
Sea_bream
Sharp-spined
Sea_bass
Bovine3
HPyV6
HPyV7
WU
KI
Common_vole
Bank_vole
Goose
Gouldian_finch
Crow
Butcherbird
Canary
Bullfinch
Budgerigar
Adelie_penguin
Elephant
Sea_lion
Badger
Vampire_bat
Mustached_bat
Nakedbacked_bat
Flatfaced_fruit_bat3
Sheep
Flying_fox_sp
Moluccan_fruit_bat3
Sulawesi_flying_fox1
Moluccan_fruit_bat2
Horseshoe_bat
Common_squirrel_monkey
Blackcapped_squirrel_monkey
Capuchin
Murine_pneumo
Multimammate_mouse
Little_brown_bat
Mouseeared_bat
Bovine1
MWPyV
HPyV10
StLPyVc
StLPyVa
StLPyVb
Chimp2s
Crabeating_macaque
Yellow_baboon1
LPV
Vervet3
HPyV9
Chimp5
Large_flying_fox
Sulawesi_flying_fox2
TSV
Orangutan_Bo
Spider_monkey
HPyV12
Hamster
Rat
MPyV
Vervet1
Red_colobus1a
Red_colobus1b
NJPyV
Chimp0b
Chimp0a
Brazilian_freetailed_bat1
Chaerephon_bat
Brazilian_freetailed_bat2
Martienssens_freetailed_bat2
Pig
Bovine2a
Bovine2b
Bonobo1
Chimp2t
Chimp2c
Chimp2a
Chimp1
MCV
Gorilla
Strawcolored_fruit_bat
Flatfaced_fruit_bat1
Sebas_shorttailed_bat
Heartnosed_bat
Martienssens_freetailed_bat1
Chimp3
Chimp4
Orangutan_Pi
Equine
Dolphin
Sea_otter
SV40a
SV40b
Vervet2
Anubis_baboon
SA12
Yellow_baboon2
Redeared_guenon
Chimp6
Chimp8
JCV
BKVIV
BKVII
BKVI
0.5
Figure S2C
